# Inhibitory effect of bovine milk osteopontin on the initial attachment of *Streptococcus mutans*

**DOI:** 10.3168/jdsc.2024-0558

**Published:** 2024-04-20

**Authors:** Hisako Ishizuka, Kazuna Ishihara, Hideo Yonezawa, Kazuyuki Ishihara, Takashi Muramatsu

**Affiliations:** 1 Department of Operative Dentistry, Cariology and Pulp Biology, Tokyo Dental College, Tokyo 101-0061, Japan; 2 Department of Microbiology, Tokyo Dental College, Tokyo 101-0061, Japan

## Abstract

We investigated the effect of bovine milk osteopontin (OPN) on the initial adhesion of *Streptococcus mutans* to hydroxyapatite. Hydroxyapatite pellets were immersed in a 5.4 µ*M* OPN solution at 37°C for 30 min. As a control, Milli-Q water was used. *Streptococcus mutans* were incubated at 37°C for 2 h on the pellets. Crystal violet staining and the quantitation of DNA were evaluated. Violet staining was observed on the hydroxyapatite pellets in the control group, but faint staining was seen in the OPN group. The amount of DNA attached to the hydroxyapatite pellet was significantly lower in the OPN group. These results suggest that initial adhesion of *S. mutans* to the hydroxyapatite is inhibited with OPN.

## Highlights

• Application of bovine milk-derived OPN on the hydroxyapatite pellets inhibits the initial adhesion of *S. mutans* on the surface of the hydroxyapatite.
• Inhibition of the initial adhesion was not different between GS5 and UA159 strains.
• Bovine milk-derived OPN would be effective for dental caries management.

## Graphical Abstract

**Figure:**
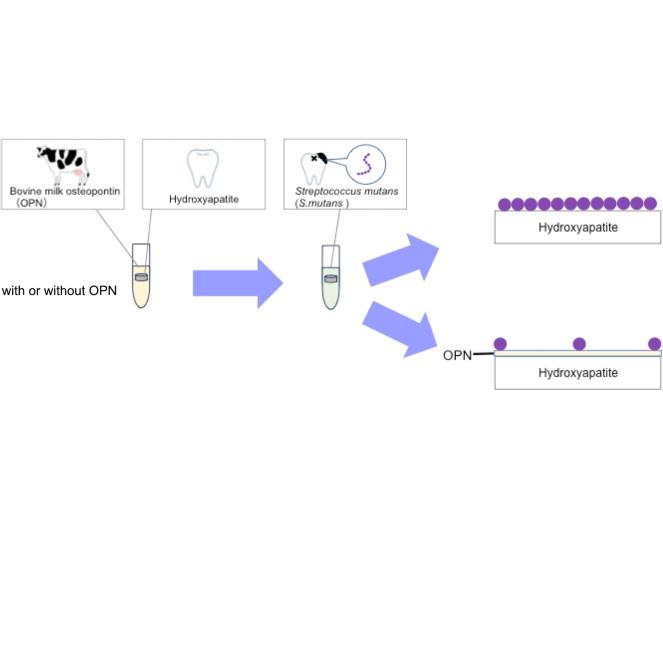
**Summary:** We investigated the effect of bovine milk osteopontin (OPN) on the initial adhesion of *Streptococcus mutans* to hydroxyapatite. Hydroxyapatite pellets were immersed in 5.4 μ*M* OPN solution at 37°C for 30 minutes. As a control, Milli-Q water was used. *Streptococcus mutans* were incubated at 37°C for 2 hours on the pellets. Results of crystal violet staining and the quantitation of DNA showed a lower quantity of *S. mutans* on the hydroxyapatite pellets in the OPN group (*P* < 0.05), suggesting that initial adhesion of *S. mutans* to the hydroxyapatite is inhibited with OPN.

One of the main causes of dental caries is acid production by cariogenic bacteria present in biofilms formed on tooth surfaces. *Streptococcus mutans* is a typical cariogenic species. *Streptococcus mutans* is involved in caries development and progression by strong attachment to tooth surfaces by protein antigen, biofilm formation by insoluble glucan (mutan) synthesis through sucrose metabolism, and induction of firm bacterial attachment to hydroxyapatite in enamel (Signoretto et al., 2014; Lin et al., 2021). Based on these findings, inhibiting the attachment of cariogenic bacteria, *S. mutans*, to tooth surfaces is considered to be an important factor in caries prevention.

Research on caries inhibition using bovine milk components has been conducted for some time, focusing mainly on their effects on demineralization inhibition/remineralization and cariogenic bacteria, *S. mutans*. As a representative example, casein phosphopeptide-amorphous calcium phosphate (**CPP-ACP**), a substance derived from casein in bovine milk, has been reported to inhibit enamel demineralization (Hamba et al., 2011). Studies focusing on the cariogenic bacterium *S. mutans* have reported that human milk and its components inhibit *S. mutans* biofilm formation (Allison et al., 2015) and that bovine milk inhibits cariogenicity and dentin demineralization of *S. mutans* (Muñoz-Sandoval et al., 2012). Furthermore, feeding probiotic milk to preschool children decreased the amount of S. *mutans* in saliva and plaque (Manmontri et al., 2020) and hydroxyapatite surfaces coated with human breast milk suppressed attachment of *S. mutans* (Wernersson et al., 2006; Niemi et al., 2009). Therefore, treatment using milk components is expected as one of the most promising caries-preventive methods clinically.

Osteopontin (**OPN**) is a noncollagenous protein discovered in bone tissue that has domains with high affinity for hydroxyapatite and calcium (Denhardt and Guo, 1993). Initially thought to be involved in osteoblast differentiation and bone remodeling, OPN has since been found to be expressed in a variety of tissues in vivo and has been shown to have a wide variety of functions including cell attachment, migration, wound healing, and cancer metastasis (Denhardt and Guo, 1993; Brown et al., 1994; Rittling and Novick, 1997; Muramatsu et al., 2002; Schack et al., 2009; Jiang and Lönnerdal, 2016). A recent report showed that OPN acts as an opsonin that attaches to bacteria and promotes phagocytosis of macrophages (Schack et al., 2009). In vivo, bovine milk contains OPN at the highest concentration (Kumura et al., 2006), and OPN from bovine milk has been shown to prevent infectious diseases in infants and to affect immune function. As the impact of OPN on the prevention of infectious diseases and immune function of infants has attracted attention, research on OPN-containing infant formulas has progressed (Lönnerdal et al., 2016; Demmelmair et al., 2017), and they have become available in Japan since 2014.

As an application of bovine milk OPN to the oral cavity aiming at caries inhibition, we have confirmed that when bovine milk OPN was applied to demineralized enamel surfaces, mineral uptake was inhibited and remineralization was suppressed, but when OPN was combined with fluoride, the remineralization effect was restored (Ishizuka et al., 2023). At the same time, research has also been conducted on the effects of bovine milk OPN on oral bacteria, leading to reports of a decrease in the bacterial attachment capacity of *Streptococcus mitis* and *Streptococcus oralis*, which are early adherent bacteria, a decrease in biofilm formation, and an increase in the pH in biofilm (Schlafer et al., 2012a,b, 2017), suggesting its application as a caries-preventive material. However, there has been no study on the effect of bovine milk OPN on the initial attachment of *S. mutans*, an important cariogenic bacterium. We hypothesize that bovine milk OPN shows the inhibitory effect on the initial attachment of *S. mutans* to the tooth surface. In this study, we investigated the inhibitory effect of bovine milk OPN applied to hydroxyapatite pellets, which are assumed to be dentine, on the initial attachment of *S. mutans*.

Hydroxyapatite pellet samples with 2 different shapes were used (square 10 mm × 10 mm × 2 mm, round ø 5 mm × 2 mm) (HOYA Technosurgical, Tokyo, Japan). Dissolved lyophilized bovine milk OPN (Sigma-Aldrich Co. LLC, St. Louis, MO) was prepared with deionized water at a concentration of 5.4 µ*M*, according to the method of Ishizuka et al. (2023). We used 2 strains of *S. mutans*, GS5 and UA159. Strains stored at −70°C were incubated at 37°C for 24 h on BD Difco Mitis-Salivarius agar medium (Becton Dickinson, Franklin Lakes, NJ), and the resulting colonies were incubated on Todd Hewitt broth (Becton Dickinson) at 37°C for 24 h. The culture medium was washed twice with PBS, then suspended in PBS to an optical density at 600 nm (OD_600_) of 1.0 and used for attachment tests.

An overview of the experimental procedure is shown in Figure 1. The samples were divided into 4 groups: (1) GS5 OPN (−) group, (2) GS5 OPN (+) group, (3) UA159 OPN (−) group, and (4) UA159 OPN (+) group, each with n = 5. The hydroxyapatite pellets of the OPN (+) groups were immersed in 1.5-mL tubes containing 5.4 µ*M* OPN solution at 37°C for 30 min. Those of the OPN (−) groups were immersed in deionized water under the same conditions. The hydroxyapatite pellets were then washed in deionized water and immersed in bacterial suspension of *S. mutans* strains GS5 or UA159 at 37°C for 2 h. Hydroxyapatite pellets immersed in *S. mutans* suspension were washed well with PBS and stained with crystal violet. The stained hydroxyapatite pellets were washed with PBS and photographed. The amount of *S. mutans* attached to hydroxyapatite pellets was assessed by measuring the amount of DNA. Hydroxyapatite pellets immersed in a suspension of *S. mutans* were washed well with PBS, and then DNA was extracted using MagExtractor -Genome- (TOYOBO, Osaka, Japan). The amount of DNA in the extracted solution was determined by quantitative real-time PCR under the following conditions: denatured at 95°C for 15 s, annealing at 55°C for 20 s, and extension at 72°C for 30 s over 30 cycles with StepOnePlus Real-Time PCR Systems (Applied Biosystems, Waltham, MA), using TB Green Premix Ex Taq II (Tli RNaseH Plus TAKARA; TaKaRa, Shiga, Japan) and the *S. mutans*-specific primer Sm F5: 5′-AGCCATGCGCAATCAACAGGTT-3′; Sm R4: 5′-CGCAACGCGAACATCTTGATCAG-3′ (Yano et al., 2002). The results obtained for each bacterial species were expressed as relative values in the OPN (+) group by setting the amount of DNA in the OPN (−) group as 1. Statistical analysis of the amount of *S. mutans* DNA attached to hydroxyapatite pellets was performed using the Mann-Whitney U test with the results obtained by quantitative real-time PCR. The significance level was set at 5%.

**Figure 1:**
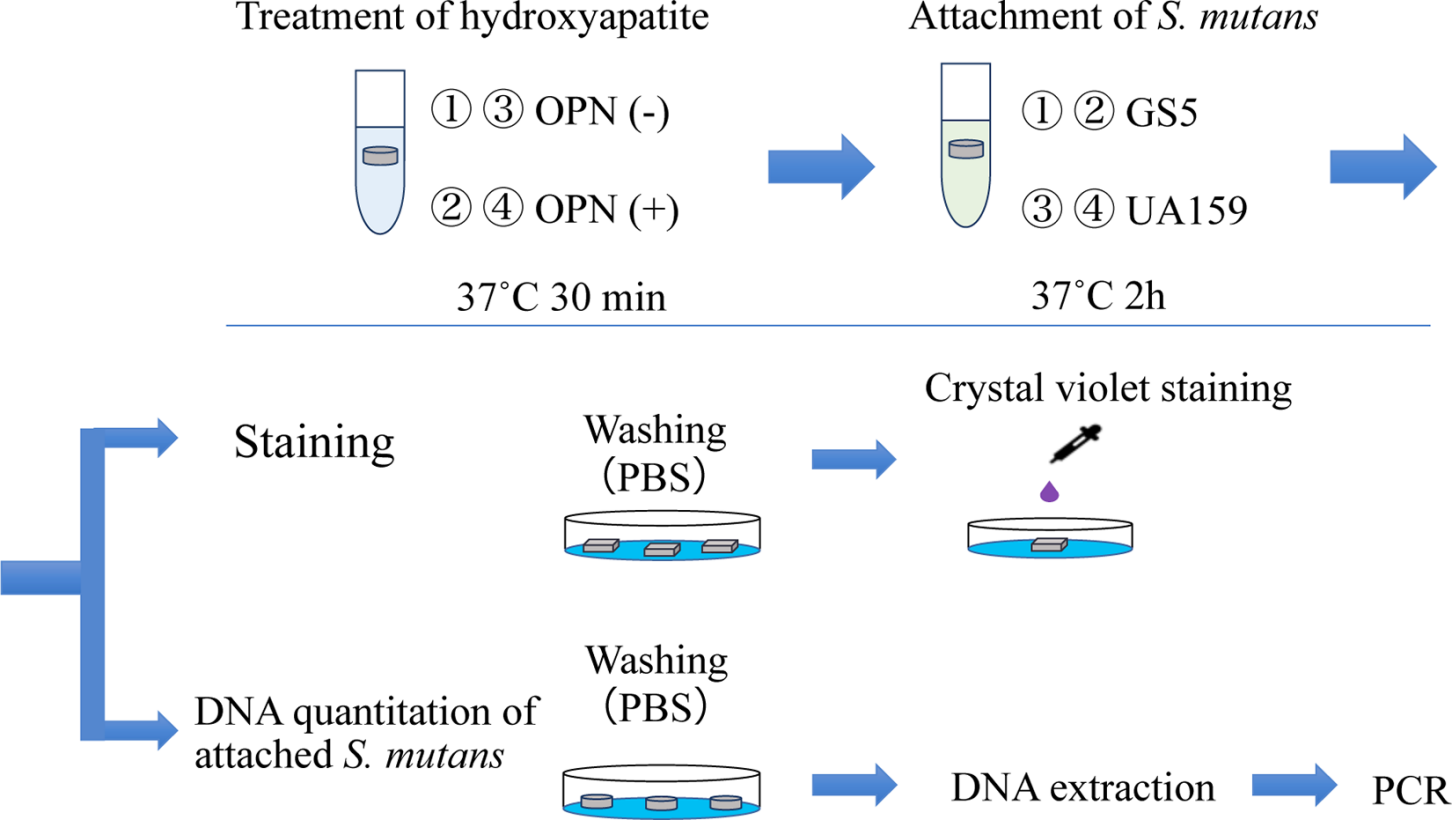
Schematic diagram showing the flow of the study procedures.

Figure 2 shows representative results of crystal violet staining in each group. The OPN (−) groups of both GS5 and UA159 strains showed staining over the entire surface of the samples (Figures 2a,b). On the other hand, among the OPN (+) groups, the GS5 OPN (+) group showed light purple staining in part of the sample margins, but no staining in the center of the samples (Figure 2c). In the UA159 OPN (+) group, no staining with crystal violet solution was observed on the entire surface of the samples (Figure 2d). The relative amount of DNA in the OPN (+) group was calculated for each strain by setting the amount of DNA in the OPN (−) group obtained by quantitative real-time PCR as 1. In the GS5 strain, the relative amount of DNA in the OPN (+) group was 0.44 ± 0.09 by setting the amount of DNA in the OPN (−) group as 1 (Figure 3a). On the other hand, in the UA159 strain, the OPN (+) group showed a relative amount of 0.21 ± 0.06 compared with the OPN (−) group (Figure 3b). Statistical analysis showed a significant difference between the OPN (−) and OPN (+) groups for both GS5 and UA159 strains at the 5% level of significance.

**Figure 2:**
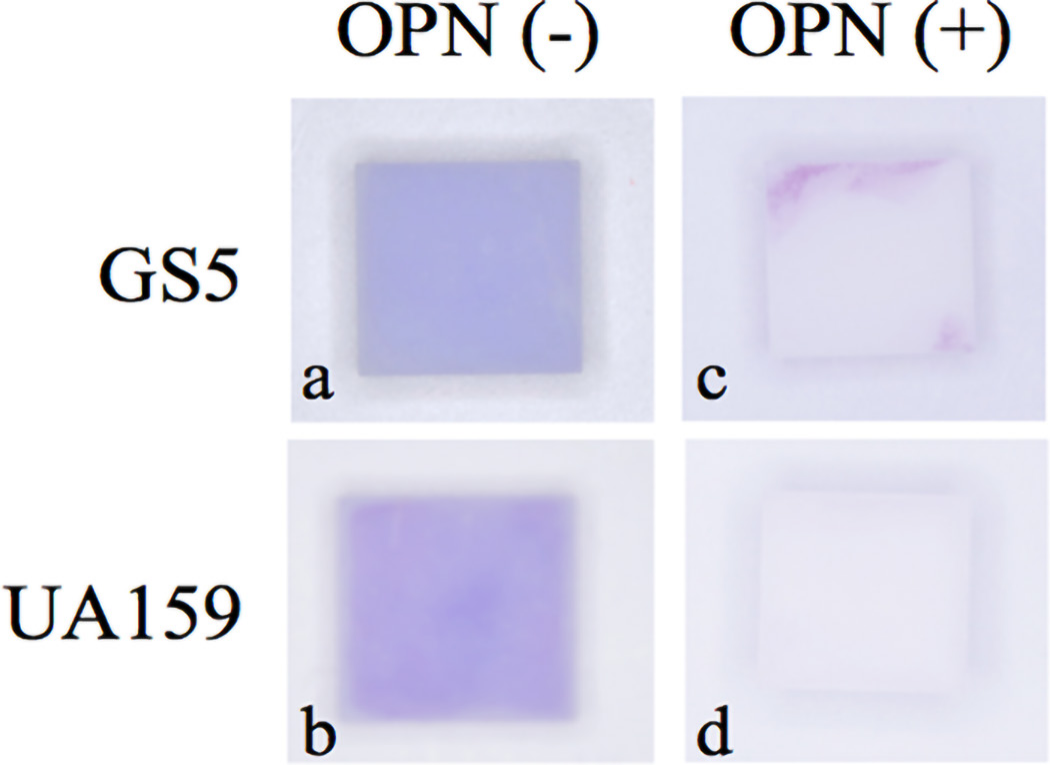
Representative figures of crystal violet staining (a) OPN (−) GS5 group, (b) OPN (−) UA159 group, (c) OPN (+) GS5 group, (d) OPN (+) UA159 group. Both GS5 and UA159 strains showed staining over the entire surface of the samples in the OPN (−) groups (panels a and b). Concerning the OPN (+) groups, the GS5 OPN (+) group showed light purple staining in part of the sample margins but not in the center of the sample (panel c). In the UA159 OPN (+) group, the entire surface of the sample was unstained with crystal violet solution (panel d).

**Figure 3:**
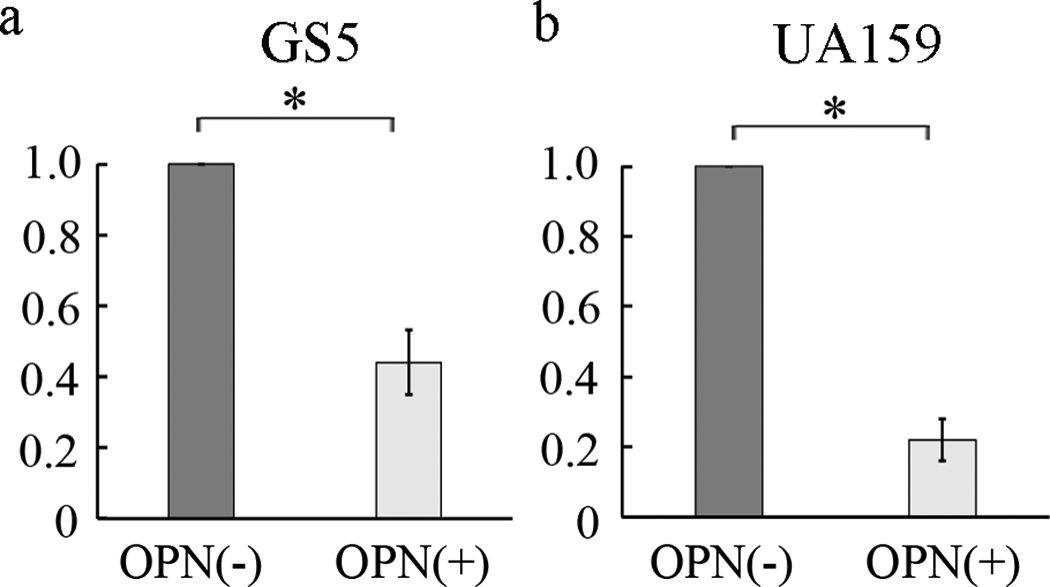
Relative amounts of *Streptococcus mutans* DNA attached to hydroxyapatite pellets. (a) GS5 strain, (b) UA159 strain. Data are presented as means ± SD. The amount of DNA in the OPN (+) group and SD when the amount of DNA in the OPN (−) group is set at 1. When the amount of DNA in the OPN (−) group was set at 1, that in the OPN (+) group was 0.44 ± 0.09 (a) for the GS5 strain and 0.21 ± 0.06 for the UA159 strain (b). There was a significant difference between the OPN (−) and OPN (+) groups for both strains. *P < 0.05.

In the present study, the initial attachment of both *S. mutans* GS5 and *S. mutans* UA159 was inhibited by treating hydroxyapatite pellets with bovine milk OPN. In previous studies about the effect of bovine milk OPN on oral bacteria, attachment of oral *Streptococcus* spp., such as *S. mitis* and *S. oralis*, was inhibited. These bacterial species are thought to adhere to enamel surfaces via lectin-like interactions and glycoconjugates, but details of the mechanism have not been elucidated (Kristensen et al., 2017). *Streptococcus mutans* is adsorbed on the pellicle via materials such as bacterial cell-surface protein antigen, and the antigen negatively charges the bacterial surface and allows bacteria to adhere to the tooth surface (Okahashi et al., 1989; Koga et al., 1990; Takahashi et al., 1993). Osteopontin is not only negatively charged but also has calcium binding sites (Denhardt and Guo, 1993). In our previous study, deposits considered to have been derived from OPN were observed by scanning electron microscopy after application of OPN to the tooth surface (Ishizuka et al., 2023). Thus, if hydroxyapatite was bound to OPN in advance as in this study, negatively charged *S. mutans* did not readily adhere to negatively charged OPN, and this may explain the inhibition of initial attachment. Although the inhibition of attachment differed between the GS5 and UA159 strains, the treatment was suggested to be effective against both strains.

Based on the results of this study, OPN derived from bovine milk can be expected to inhibit the attachment of cariogenic bacteria, *S. mutans*, to the tooth surface, and may be useful as a caries-preventive material in the future. In clinical application, its high biological safety and impact on oral flora should be considered. In terms of biological safety, OPN is abundant in human milk (Schack et al., 2009), has already been added to infant formula, and is commercially available (Jiang and Lönnerdal, 2016). Regarding the effect on oral commensal bacteria, there is a report (Kristensen et al., 2022) that it can inhibit tooth surface attachment of bacteria without causing harmful side effects on the resident flora, thus its application to the oral cavity should not be a problem. In the actual application based on the results of this study, since pellicles exist on the tooth surfaces, OPN should be applied after removing the pellicles by tooth polishing or other means. This method is similar to the in vitro method of Ishizuka et al. (2023), but in this case, the concomitant use of fluorides is desirable as the remineralization effect of saliva is also suppressed.

Many studies have evaluated the biofilm formation ability of *S. mutans* using hydroxyapatite pellets. However, in recent years, some studies have begun to evaluate the surfaces of hydroxyapatite pellets after treatment with saliva for simulation of the actual oral environment (Wernersson et al., 2006; Spengler et al., 2017). In particular, studies have shown that *S. mutans* protein antigen adheres to the pellicle (Okahashi et al., 1989; Koga et al., 1990; Takahashi et al., 1993), and since the presence of saliva has been reported to facilitate *S. mutans* attachment (Shimotoyodome et al., 2007), we are considering future experiments in which hydroxyapatite pellets are treated with saliva and then with bovine milk OPN, to simulate the oral environment.

In this study, we investigated the initial attachment of *S. mutans* strains GS5 and UA159 by applying bovine milk OPN to hydroxyapatite surfaces. In conclusion, treating hydroxyapatite surface with bovine milk OPN inhibited the initial attachment of both *S. mutans* GS5 and UA159 strains.
